# The Impact of Public Health Events on COVID-19 Vaccine Hesitancy on Chinese Social Media: National Infoveillance Study

**DOI:** 10.2196/32936

**Published:** 2021-11-09

**Authors:** Zizheng Zhang, Guanrui Feng, Jiahong Xu, Yimin Zhang, Jinhui Li, Jian Huang, Babatunde Akinwunmi, Casper J P Zhang, Wai-kit Ming

**Affiliations:** 1 Jinan University-University of Birmingham Joint Institute Jinan University Guangzhou China; 2 School of Mathematics, College of Engineering and Physical Sciences University of Birmingham Birmingham United Kingdom; 3 School of Medicine Jinan University Guangzhou China; 4 School of Journalism and Communication Jinan University Guangzhou China; 5 Singapore Institute for Clinical Sciences Agency for Science, Technology and Research Singapore Singapore; 6 Department of Epidemiology and Biostatistics School of Public Health, Faculty of Medicine Imperial College London London United Kingdom; 7 Department of Obstetrics and Gynecology Brigham and Women’s Hospital Boston, MA United States; 8 Center for Genomic Medicine Massachusetts General Hospital, Harvard Medical School Harvard University Boston, MA United States; 9 School of Public Health The University of Hong Kong Hong Kong China (Hong Kong); 10 Department of Infectious Diseases and Public Health Jockey Club College of Veterinary Medicine and Life Sciences City University of Hong Kong Hong Kong China (Hong Kong); 11 School of Public Policy and Management Tsinghua University Beijing China

**Keywords:** COVID-19, vaccine, hesitancy, social media, China, sentiment analysis, infoveillance, public health, surveillance, Weibo, data mining, sentiment, attitude

## Abstract

**Background:**

The ongoing COVID-19 pandemic has brought unprecedented challenges to every country worldwide. A call for global vaccination for COVID-19 plays a pivotal role in the fight against this virus. With the development of COVID-19 vaccines, public willingness to get vaccinated has become an important public health concern, considering the vaccine hesitancy observed worldwide. Social media is powerful in monitoring public attitudes and assess the dissemination, which would provide valuable information for policy makers.

**Objective:**

This study aimed to investigate the responses of vaccine positivity on social media when major public events (major outbreaks) or major adverse events related to vaccination (COVID-19 or other similar vaccines) were reported.

**Methods:**

A total of 340,783 vaccine-related posts were captured with the poster’s information on Weibo, the largest social platform in China. After data cleaning, 156,223 posts were included in the subsequent analysis. Using pandas and SnowNLP Python libraries, posts were classified into 2 categories, positive and negative. After model training and sentiment analysis, the proportion of positive posts was computed to measure the public positivity toward the COVID-19 vaccine.

**Results:**

The positivity toward COVID-19 vaccines in China tends to fluctuate over time in the range of 45.7% to 77.0% and is intuitively correlated with public health events. In terms of gender, males were more positive (70.0% of the time) than females. In terms of region, when regional epidemics arose, not only the region with the epidemic and surrounding regions but also the whole country showed more positive attitudes to varying degrees. When the epidemic subsided temporarily, positivity decreased with varying degrees in each region.

**Conclusions:**

In China, public positivity toward COVID-19 vaccines fluctuates over time and a regional epidemic or news on social media may cause significant variations in willingness to accept a vaccine. Furthermore, public attitudes toward COVID-19 vaccination vary from gender and region. It is crucial for policy makers to adjust their policies through the use of positive incentives with prompt responses to pandemic-related news to promote vaccination acceptance.

## Introduction

At the end of 2019, the first case of COVID-19 was reported in Wuhan, China. The disease spread rapidly throughout China, after which it soon evolved into a global pandemic. By the end of May 2021, the total number of confirmed cases globally exceeded 100 million, and the cumulative number of deaths was >3 million with a mortality rate of approximately 2.07% [1]. Although the rate was lower than that of severe acute respiratory syndrome coronavirus 1 and Middle Eastern respiratory coronavirus (9.5% and 34.4%, respectively), it cannot be ruled out that COVID-19 has stronger transmissibility than either one of those viruses [2,3]. The rapid spread of COVID-19 has brought unprecedented challenges to each country worldwide in terms of social, economic, cultural, and political aspects.

Vaccination is considered the most effective and safest way to provide immunity against new infectious diseases. According to statistics, the current kinds of vaccines worldwide can save more than 3 million lives related to >20 diseases every year [4]. To control the worldwide spread of COVID-19, a call for global vaccination against COVID-19 is required [5]. In mid-March 2020, China’s recombinant COVID-19 vaccine was approved, and clinical trials were initiated [6]. Thus far, at least 13 different COVID-19 vaccines have been put into use throughout the world, including the Sinopharm COVID-19 vaccine [7]. However, with the continuous development of the internet worldwide, the antivaccine campaign is also spreading rapidly through social media platforms, thus causing a threat to optimal global vaccine delivery [8-10].

Social media has played a key role in information dissemination during the COVID-19 pandemic. Through social media, important epidemic-related information can be easily disseminated, and people across the world can quickly obtain relevant disease-related information, participate in the discussions, and express their own views about COVID-19 [11,12]. In the meantime, misinformation, defined as erroneous or incorrect information, has also been widely spreading during the pandemic [13]. Although misinformation about COVID-19 is posted more than evidence-based information on social media [14], scientific information has had more reposts than the false information [14], and the platforms have responded to much misinformation identified by fact-checkers [15].

Weibo is one of the representative social media platforms with most users in China, which has more than 500 million active users and more than 700 billion views [12,16-19]. It has become one of the primary social platforms for Chinese internet users to disseminate and acquire health information [20]. Up to June 2020, China had nearly 1000 million netizens, accounting for 67% of all Chinese citizens [21,22]. According to the 2020 annual report released by Weibo, users checked COVID-19–related information 16.1 billion times every day during the outbreak [23]. Particularly regarding the COVID-19 vaccine, more than 100,000 Weibo users participated in the discussion with a cumulative reading of more than 500 million times [24]. Given its popularity and the massive information contained within the site, Weibo can be considered an appropriate data source to investigate the public attitudes toward the COVID-19 vaccine.

For sentiment analysis during public health emergencies, many studies have used web crawlers, text-mining, and other technologies to collect information regarding a variety of public opinions from the internet [25]. In addition, some studies have used the web text data in accordance with different phases, classified these data on the basis of the theme and emotion [26,27], and adopted various visualization tools to investigate the public sentiment, thus proving that the social media can be applied to measure the public attention toward public health emergencies [27]. With the ongoing COVID-19 pandemic worldwide and successful entry of its related vaccines on the market, some studies have begun to focus on the information on social media to analyze the acceptance of vaccines by the public, emphasizing that the public’s attitude on health issues are strongly influenced by social media [28].

However, many studies have not yet analyzed the sentiments of the Chinese population nationwide through their statements on domestic social media sites such as Weibo. While a number of studies have been conducted abroad on social media platforms, such as Twitter and Facebook [9,29-31], we do not yet clearly know the current sentiments and attitudes of the population toward COVID-19 vaccination in China. Furthermore, only a few studies have investigated the relationship between the social media context and public sentiment toward vaccination [32]. Clear and concise sentiment analysis of textual information on Weibo will not only improve the monitoring of public opinions on the internet but also effectively allow for the application of the results of emotional psychology studies to provide early warnings of unusual occurrences. The study of such psychological indicators is a very important guide for government policies at this particular stage [33-37] and would enable national governmental departments to better understand the attitude of the public toward vaccination, thus advancing collaboration with multiple parties more effectively to increase the vaccination rate of COVID-19.

This study aimed to investigate the public sentiment of COVID-19 vaccines and to evaluate gender and regional variations in this sentiment. The feasibility of social sentiment analysis on the basis of web-based data of hot-spot events and whether the same approach can be used in the future to keep track of public opinions on the internet during the vaccination period was assessed. The aim of this process was to provide a realistic grasp of the dynamic psychology of the public and highlight the leading role of the national government departments. The study also highlights the essential role of national governmental departments in moderating public sentiment through social media.

## Methods

### Methods Overview

Based on the public nature of the Weibo platform, this study used Python for data mining and sentiment analysis of the resulting text to crawl and analyze public comments published by Weibo users on the issue of COVID-19 vaccination, thus allowing the identification of the sentiment tendencies of the resulting text.

### Data Collection

#### Processing

Python 3.9.2 (Python Software Foundation) [38] and related libraries were utilized to simulate logging and then capture the required data. The data obtained containing the identifier (ID) of the post, the context of the post, the post time, the repost times, the number of “likes,” the gender of the person posting, the location of this person, and the posting person’s birthday were saved as multiple csv files. Owing to the anticrawler mechanism of Weibo, outliers beyond the setting date or keyword ranges were excluded.

#### Inclusion Criteria

Data were captured from the search results of Weibo with the keyword “COVID-19 Vaccine (新冠 疫苗)” between October 18, 2020, and May 15, 2021 (inclusive of both dates). As the general search criteria, the captured posts could refer to any approved COVID-19 vaccines globally. However, the search results may tend to the vaccine that is available in China. Since the availability of the vaccine to the Chinese public can be dated back to mid-October 2020 [39], the chosen time period is believed to cover the process from vaccine development to the mass vaccination scheme.

#### Exclusion Criteria

Given that the study focused on the public opinion in China, any texts written in languages other than Chinese and those from users whose locations were outside China were excluded. Any posts consisting of only symbols or numbers were also excluded.

### Data Cleaning

#### Text Cleaning

First, the text contains no Chinese characters, namely posts written in other languages, or posts consisting of only symbols or numbers were removed. Then, posts with missing information, such as location or date, duplicated posts and those from public accounts were also removed [40].

#### Relevance-Based Cleaning

Because of the specific writing style of social media, the relevance between the context and study topic is a vital issue to be considered [41]. In this part, a “base text” is set to describe the proven determinants of COVID-19 vaccine acceptance in China [42] and compared with each crawled post to obtain their similarity. Cosine similarity, which is conceived as a powerful approach in natural language processing (NLP), was performed to measure the similarity between the crawled post and base text as formulated in the following model:



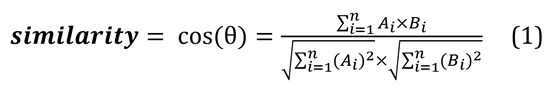



in which *A_i_* and *B_i_* are the *i*th item of the word frequency vector of the extracted keyword list via term frequency–inverse document frequency (TF–IDF) from the base text and crawled posts, respectively. After a trail contained 1000 randomly chosen posts, a threshold of 0.025 was set to distinguish the relevant post from the irrelevant ones, which attained an accuracy rate of 94.1% (941/1000). The model was then applied to the data set, and irrelevant posts were excluded.

#### Sentiment Analysis

Sentiment analysis is an NLP-based method to detect subjectivity in text, extracting and classifying opinions and sentiments [43]. SnowNLP [44], which is a specialized Python library for Chinese language processing and has been used in social media text mining for medical studies, especially COVID-19–related studies, given its feasibility and accuracy [21,45-47], was used to perform sentiment analysis.

In total, 15,000 randomly chosen posts were annotated manually, each of them was coded by 2 researchers, one of whom annotated independently and the other double-checked, of which 12,000 and 3000 posts were randomly split into the training and test sets. The training set included 9084 positive and 2916 negative posts (“neutral” was not used as a category owing to its limited research significance [45]).

**Figure 1:**
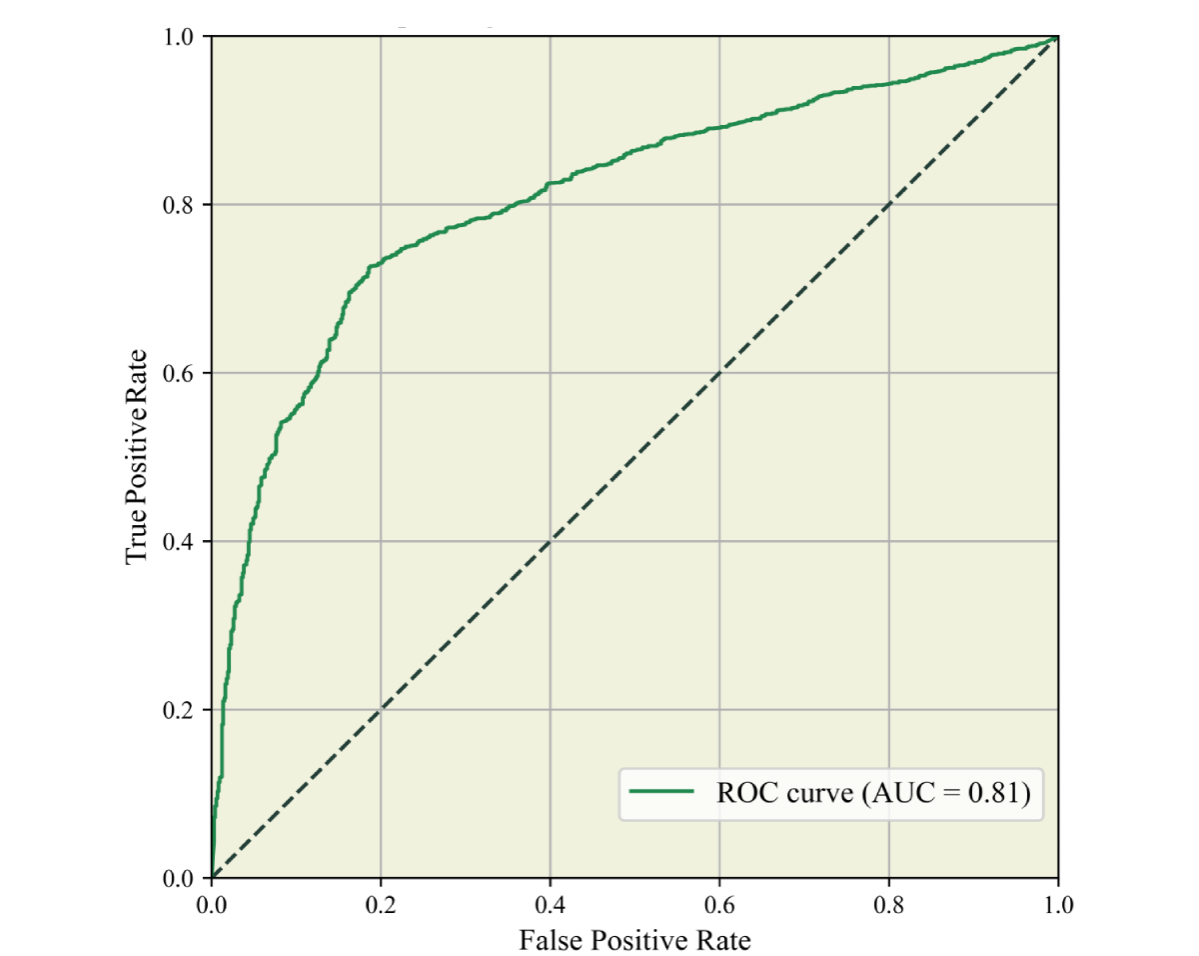
Receiver operating characteristic curve of the fitted SnowNLP model. AUC: area under the receiver operating characteristic curve; ROC: receiver operating characteristic.

The process of SnowNLP includes word segmentation, stop word removal, and naïve Bayes classification. The key model is shown below:



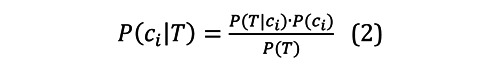



where:

*P*(*T*) = *P*(*T*|*c*_1_) · *P*(*c*_1_) + *P*(*T*|*c*_2_) · *P*(*c*_2_)

in which *T* is the eigenvector of the text, and *c*_i_ is the *i*th emotion category, in this case, positive and negative. The posts are divided into those with probabilities higher than the threshold (positive category) or negative otherwise. Given the unbalanced distribution of labels in the training set, a receiver operating characteristics (ROC) curve was introduced [48] to evaluate the model (Figure 1). The area under the ROC curve (AUC) was then computed to measure the outcome of the classifier. After training, the threshold was set as 0.5889, for which the AUC yielded 0.81.

After training the model, the emotions in the posts were computed. The sentiment score, *S* was calculated using the following equation:



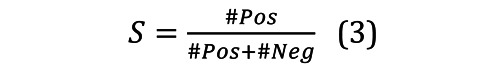



in which #*Pos* and #*Neg* are the number of positive and negative posts, respectively. A sentiment score ranged from 0 to 1, indicating the most negative to the most positive.

## Results

A total of 340,783 posts, including both original and reposted posts, and user-related information were retrieved. After cleaning, 156,223 posts were included in the analysis. According to the statistics, more female than male posters were noted, and more positive than negative posts were identified. Table 1 shows the number of posts and users.

**Table 1:** Descriptive statistics of total posts.

Topics		Posts or users, n (%)
**Users (n=98,600)**		
	Male users	45,812 (46.5)
	Female users	52,788 (53.5)
**Total posts (n=156,223)**		
	Posts from male users	84,353 (54.0)
	Posts from female users	71,870 (46.0)
	Positive posts	93,660 (60.0)
	Negative posts	62,563 (40.0)

**Figure 2:**
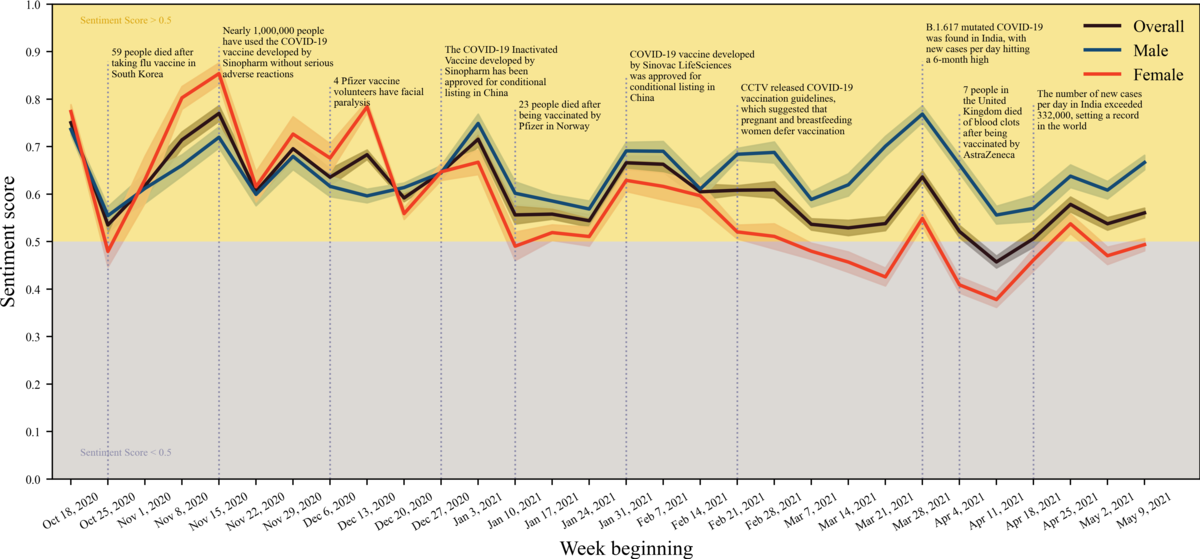
The variation in public sentiment toward COVID-19 vaccines over time between male and female users (a higher sentiment score indicates higher positivity toward COVID-19 vaccines).

As shown in Figure 2, the overall public positivity tends to fluctuate over time. The decline in positivity was consistent with the reported information about the side effects of the COVID-19 vaccine or other vaccines in general. In the week after October 18, 2020, a total of 59 people in South Korea were reported to have died after receiving the influenza vaccine, and at the same time, a rapid decline in the overall user’s positivity for the COVID-19 vaccine occurred a short time after that report. In terms of gender, both men and women presented positive attitudes about the COVID-19 vaccine across most of the study periods, and the fluctuation patterns of the emotional score between male and female users is generally similar. Interestingly, although the trend of male and female emotional fluctuations was generally consistent, the overall positivity was weaker among female users than among male users most of the time. During the period from February 21 to March 21, 2021, China Central Television (CCTV) announced that pregnant and lactating women should postpone vaccination. Unlike male users, female user positivity for the COVID-19 vaccine decreased rapidly during this week (February 21-27, 2021) but began to rise again in the week.

**Figure 3:**
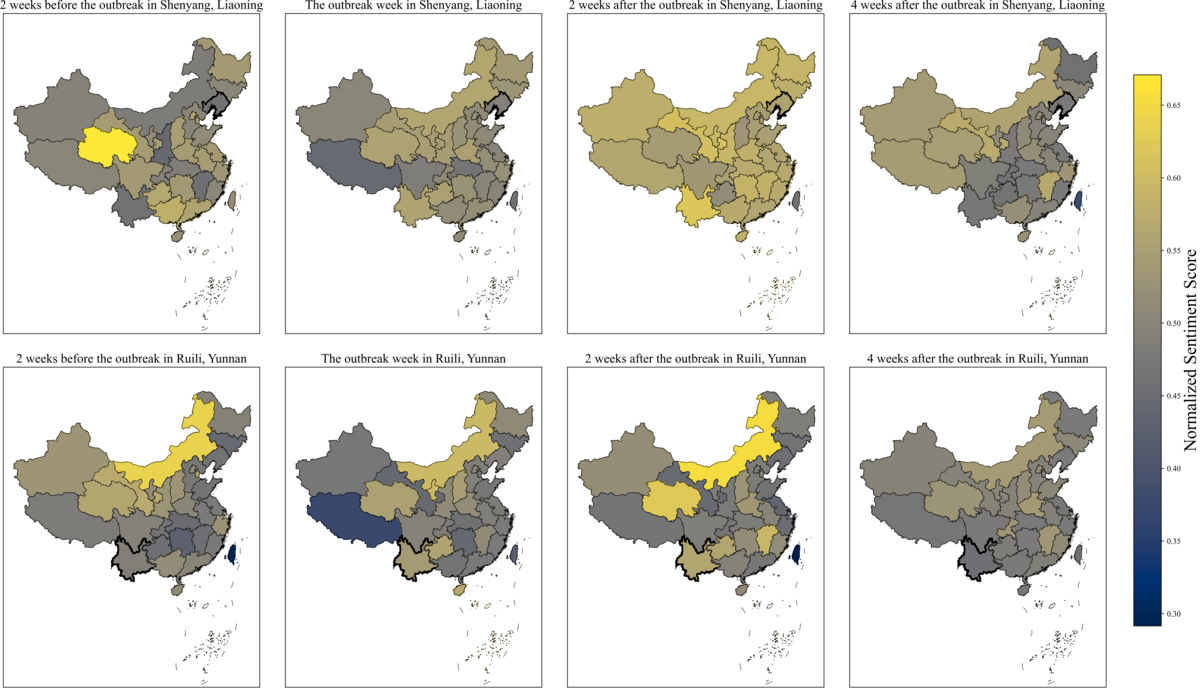
Variation in public positivity toward COVID-19 vaccines before and after the outbreak period in Ruili and Shenyang (the boundaries of provinces with the outbreak are highlighted in bold blacked lines).

Considering the 2 outbreaks in Shenyang, Liaoning Province, in January 2021 and Ruili, Yunnan Province, in March 2021, we present the heat map of the normalized sentiment score across all regions and provinces of China in heat maps by focusing on the period from 2 weeks before the outbreak to 4 weeks after the outbreak (Figure 3). Since the outbreak in these 2 regions, the sentiment about vaccination in this region and its surrounding regions had increased significantly, whereby vaccination positivity gradually declined 2 weeks after the outbreak was reported. In terms of the outbreak in Shenyang (upper panels in Figure 3), vaccination positivity increased not only in its own province but also in Northeast China and even throughout the country. Similarly, after the outbreak was reported in Ruili, Yunnan Province, the sentiment toward vaccination in Guizhou Province, a neighbor of Yunnan, also increased significantly.

## Discussion

### Principal Findings

COVID-19 vaccine hesitancy is a worldwide phenomenon and is a crucial issue to be solved in the fight against the pandemic. In China, we found that gender-specific emotional responses to vaccines could be influenced by various major public events over time, and the degree of influence varied by gender with women as the more vaccine-hesitant group. Furthermore, public positivity changed significantly in the weeks before and after the COVID-19 outbreak. A fairly substantial body of previous research demonstrating that women experience and express more intense emotion than men with regard to both positive and negative emotions can be found [49-51]. Recent media studies further indicate that female users are more likely to seek emotional support from web-based communities, while male users tend to provide information-related help [52,53]. Moreover, the difference in vaccine acceptance between genders has been reported in some previous studies focusing on flu vaccines, for which vaccine acceptance is greater in men than in women [54-56]; these findings also support our results. This study confirmed the emotional tendency of male and female users toward COVID-19 vaccines on social media, thus extending the literature on gender differences to the specific context of public health events. The findings also inspire policy makers for refined strategies in negative sentiment management. Interestingly, although previous studies show that women are more vaccine-hesitant [54-56], some studies have reported that vaccine coverage may be higher in women than in men [54]. The reason for this difference could result from women visiting preventive health care services and physicians more frequently [57]. Therefore, the policy maker should pay more attention to female communities’ sentiments concerning major public health events and at the same time monitor the vaccination coverage to provide the in-time responses.

We found that the emotional tendencies of the pubic are dynamic, and positive and negative tendencies exist with respect to expressed emotional tendencies by the public. Every time a vaccine-related adverse event occurs, it may cause a decline in positive sentiment among Chinese internet users on Weibo, which is highly contagious on social media. Meanwhile, spreading false and appalling information on Weibo, which may bring about feelings of depression and anxiety for certain groups of people [58,59]. Therefore, when a COVID-19 emergency occurred in their particular region, people’s sense of fear and self-protection led to increased positive emotions toward vaccination. Interestingly, during the outbreak in Shenyang, Liaoning, the positivity increased in this epidemic region and throughout the whole country. Even though the scenario could have resulted from the outbreak in other provinces in that period, we cannot rule out the possibility that regional outbreaks may affect national positivity. Furthermore, after the outbreak of the epidemic in Shenyang, Liaoning Province, Yunnan Province was the province with one of the most significant increases in vaccination positivity. Therefore, the public positivity of COVID-19 vaccines in previous epidemic regions may have a retrospective effect on vaccination positivity. As the situation of the outbreak improved, people began to gradually decrease the release of positive emotions again owing to decreased vigilance. Future studies should consider the use of social media to guide the public sentiment after the epidemic outbreak is over.

Although other emerging studies have investigated the intention toward COVID-19 vaccination using methods such as questionnaires, limitations such as the existence of some bias by inferring the perceptions and attitudes of the group with only a small sample still exist [60-64]. Sentiment analysis through the use of big data offers a more direct way to monitor the emotion of the citizens. Further studies should focus on the relationship between the positivity and the case rate growth or death rate.

With the popularity of the internet and economic development, social media has become a medium for people to express their emotions and opinions. For government officials and public health professionals, understanding public sentiment is critical to develop policies for infectious disease prevention and control and health care resource allocation. In the context of a global COVID-19 outbreak, the vaccine is an important measure to establish herd immunity against COVID-19 in an open border setting [65]. Therefore, understanding public sentiment about vaccines is an effective way for the government to promote COVID-19 vaccination in a rational and orderly manner. Exploring the factors and behaviors that influence positivity between different genders and different regions through the use of internet-based data can provide relevant information for government departments that are trying to assist in decision-making and providing health services. It also reminds relevant departments to establish public opinion and sentiment monitoring networks to understand the dynamics of public attitudes toward vaccines, predict changes in sentiment, and plan vaccine production and resource allocation rationally. This process is crucial for the government to better understand public sentiment through social media and to convey information accurately and timely, which will also answer vaccine-related queries and increase vaccination motivation.

Sentiment analysis can reveal differences between cities and regions, and when combined with current COVID-19 vaccine postings on social media and dynamic microblog postings based on geolocation data, can be used as a decision support point for government agencies. This type of analysis can also provide effective and real-time recommendations to government agencies that are based on the average number of microblogs per city and region and emotional tendencies; if this number is well above a significant peak, the information can be quickly reported to official agencies. Our text sentiment analysis tool can be an extension of this research, capturing the relevant information needed in real-time.

### Limitations

This study has some limitations. First, our data collection was conducted only on one social media platform, Weibo. The opinions of those who did not use Weibo were not included. Second, the coding process was not completely independent, which may cause bias in the training process. Third, the gender and location information were self-reported by the users, which is a common issue in studies based on social media such as twitter [66]. Fourth, owing to the anticrawler mechanism of Weibo, a small proportion posts randomly lost during crawling. Fifth, sentiment may not be the only factor affecting vaccination acceptance. Local governments may take advantage of social media in promoting vaccination, but other challenges such as misinformation and the allocation of vaccines still exist.

### Conclusions

The public opinion is closely related to public health events in China. When positive news about COVID-19 vaccines occurs, the public will be more positively sentimental about the vaccine and vice versa. This sentimental reaction appears to be gender-specific, by which men tend to be more open-minded than women. In terms of regional differences, the positivity of a province and its surrounding (and even the whole country) in which a pandemic occurs, was shown to increase and then decrease back to normal after 2-4 weeks. It is crucial for the government to adjust vaccination policies promptly in response to the public health events to promote massive vaccination via dynamic monitoring public sentiments.

## Abbreviations

AUC: area under the receiver operating characteristic curve
CCTV: China Central Television
NLP: natural language processing
ROC: receiver operating characteristic
TF–IDF: term frequency–inverse document frequency
